# Landscape of current practices and future perspectives in living donor kidney donation in Europe: proceedings of a pan-European symposium by the European kidney transplant association section of the European Society for Organ Transplantation

**DOI:** 10.3389/ti.2026.16548

**Published:** 2026-07-01

**Authors:** Andreas Kousios, Marije Baas, Anna Manonelles, Fernanda Ortiz, Elvana Rista, Rachel Thomas, Carmen Lefaucheur, Emin Baris Akin, Lorna Marson

**Affiliations:** 1 School of Medicine, European University Cyprus, Nicosia, Cyprus; 2 Nefrontida Medical Centre, Nicosia, Cyprus; 3 Department of Nephrology, Radboud University Medical Center, Radboud Universiteit, Nijmegen, Netherlands; 4 Renal Transplant Unit, Nephrology Department, Bellvitge University Hospital, Barcelona, Spain; 5 Abdominal Unit, Nephrology, Helsinki University Hospital, Helsinki, Finland; 6 Department of Nephrology, Dialysis, and Kidney Transplantation, Hygeia International Hospital, Tirana, Albania; 7 Edinburgh Transplant Centre, Royal Infirmary of Edinburgh, Edinburgh, United Kingdom; 8 Saint Louis Hospital, Assistance Publique- Hopiteux de Paris and Université Paris Cité, Paris, France; 9 Department of General Surgery, Demiroglu Bilim University and Group Florence Nightingale Hospital, Istanbul, Türkiye

**Keywords:** cross-border organ exchange, donor evaluation and safety, equity in transplantation, living donor kidney transplantation, transplant registry harmonisation

## Abstract

Living donor kidney transplantation (LDKT) offers superior outcomes for most patients with end-stage kidney disease (ESKD), yet its uptake across Europe remains highly variable. This proceedings article summarizes key themes from a pan-European symposium held in November 2025 in Prague, organized by the European Kidney Transplant Association (EKITA) in collaboration with the DESCaRTES Working Group. Discussions highlighted substantial heterogeneity in LDKT activity across Europe, driven by differences in healthcare capacity, legal frameworks, donor evaluation practices, and access to kidney exchange programmes. Marked inequities persist between regions, particularly in the Balkans and Western Balkans, for women those who are socioeconomically disadvantaged, ethnic minority populations, paediatric and elderly patients and individuals with obesity. The symposium identified wide variation in donor selection criteria, risk assessment, informed consent practices, and long-term donor follow-up, despite existing international guidelines. Emerging strategies to address these challenges include harmonisation of donor evaluation and consent, expansion of paired and cross-border kidney exchange programmes, increased use of unspecified kidney donation, and adoption of innovative surgical and immunological approaches to safely broaden donor eligibility. Advances in outcome measurement, including validated surrogate endpoints, machine learning methods, and integrated, harmonised transplant registries, were discussed as critical tools to improve quality, transparency, and research efficiency. Collectively, the proceedings underscore the need for coordinated clinical, policy, and data-driven solutions to reduce inequities and unlock the full potential of LDKT across Europe, with implications for international transplant practice.

## Introduction

Living donor kidney transplantation (LDKT) provides the best outcomes for most patients with end-stage kidney disease (ESKD), particularly when performed pre-emptively before dialysis initiation [1, 2], yet its uptake across Europe remains highly variable. Differences in clinical practice, legal frameworks, donor evaluation, and access to kidney exchange continue to drive inequities in LDKT across Europe. Reflecting the need for coordinated European action, EKITA has identified promotion of LDKT as a strategic priority for 2026–2027. This proceedings article summarizes key themes from a pan-European symposium held in Prague in November 2025, organized by EKITA and the DESCaRTES Working Group, highlighting current practice, unmet needs, and future strategies to improve equitable access to LDKT in Europe.

### Variation in living donation across Europe

**FIGURE 1:**
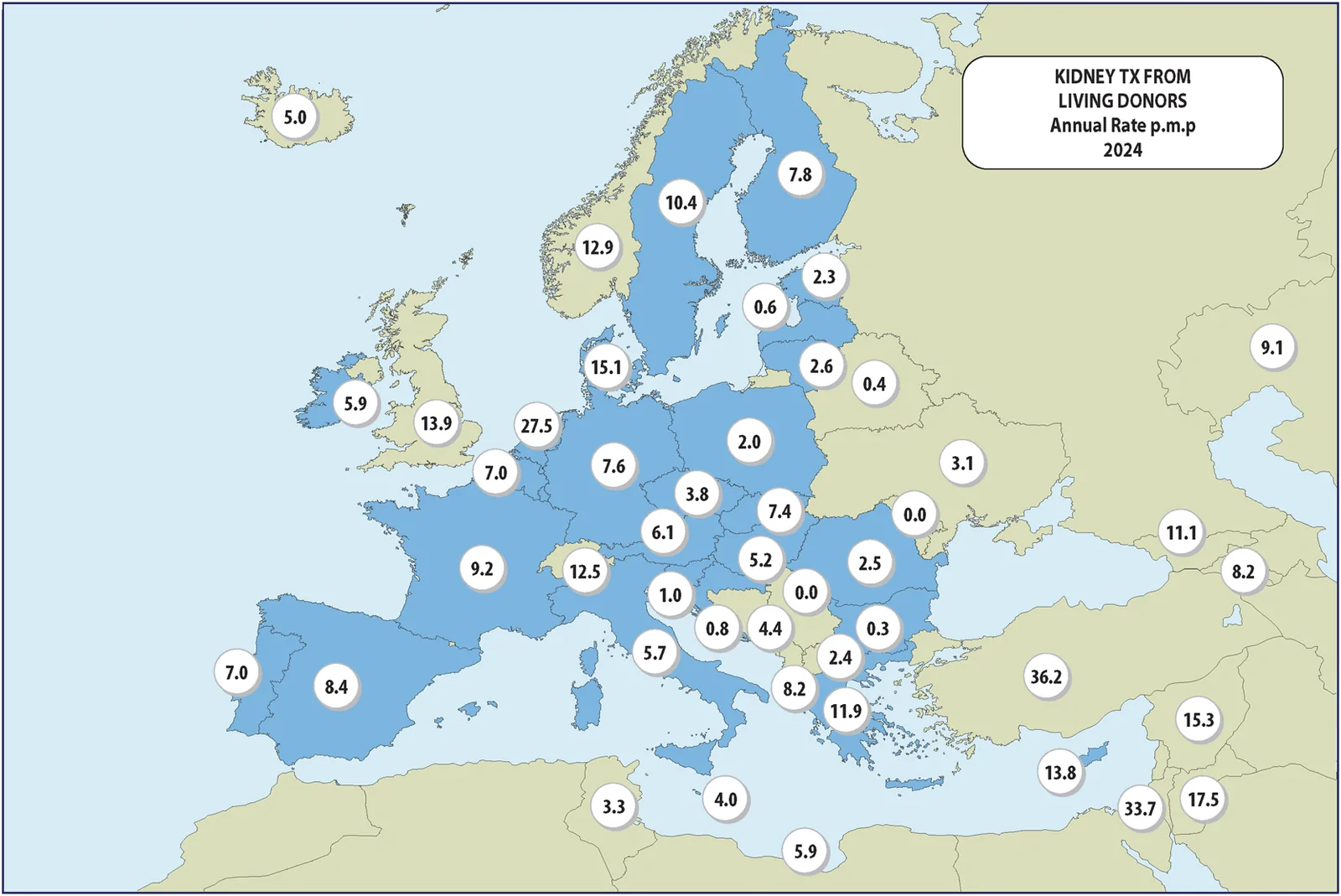
Living donor kidney transplantation rates (per million population) across Europe in 2024. Source: European Directorate for the Quality of Medicines and Healthcare (EDQM). Reproduced from EDQM/Council of Europe and Organización Nacional de Trasplantes (2025), Newsletter Transplant - International figures on donation and transplantation 2024, vol. 30, available at https://go.edqm.eu/nltransplant.

Across the continent, LDKT activity remains unevenly distributed. The overall living donor transplant rate is approximately 13 per million population (pmp), compared with ∼27 pmp for deceased donor transplantation, with extremes ranging from negligible to non-existent activity in some countries such as Moldova, Slovenia and Serbia with ∼0 pmp rates, to ∼39 pmp in Turkey. Countries such as the Netherlands and parts of Scandinavia demonstrate high LDKT activity, supported by mature national and kidney exchange programs, whereas many Western, Central, and Southern European countries, including Poland, France, Italy, and much of Central Europe, exhibit comparatively lower rates. In Central Europe, 2024 LDKT rates ranged from 0.95 pmp in Slovenia and 2.18 pmp in Poland to 7.59 pmp in Germany, despite well-developed transplant infrastructures (Figure 1) [3].

Disparities are even more pronounced in the Balkans and Western Balkans, where living donation often compensates for underdeveloped deceased donor programs. Living donation rates range from 0.3 to 0.5 pmp in Bulgaria to 2.8–2.9 pmp in Romania, with Greece representing a regional outlier at approximately 10 pmp. In the Western Balkans, Albania (∼9.3 pmp), Bosnia and Herzegovina (∼9.7 pmp), and North Macedonia (∼9 pmp) rely predominantly on living donation, whereas Serbia demonstrates critically low activity and Kosovo lacks a domestic transplant program. Overall, fewer than 25% of patients with ESKD in the Western Balkans receive a kidney transplant, compared with ∼40% across the European Union, highlighting persistent inequities driven by healthcare capacity, cultural factors, and limited regional integration.

### Analysis of European practices in living kidney donor evaluation

Thorough assessment of living kidney donors is essential to ensure donor safety. Despite the availability of multiple national and international guidelines for living donor evaluation [4–6], substantial variation in clinical practice persists across Europe. In the first Europe-wide survey conducted by the DESCaRTES and EKITA working groups (124 centers in 16 countries, representing ∼45% of European centers and ∼3,700 living kidney donations annually), marked heterogeneity was observed in donor evaluation and follow-up practices (Table 1) [7]. Low-consensus areas included kidney function assessment, nephrolithiasis, albuminuria, microscopic haematuria, and BMI thresholds, highlighting the need for harmonised donor assessment criteria across Europe (Table 1). Practice variation reflects not only differences between countries but also centre-level policies and organisation. For example, a recent UK survey demonstrated marked variation in donor acceptance criteria, evaluation timelines, and follow-up practices despite a shared healthcare system and excellent outcomes [8].

**TABLE 1:** Summary table of key findings Living Kidney Donation Practices in Europe: A Survey of DESCaRTES and EKITA Transplantation Working Groups (ref).

Domain	Key finding	Interpretation/Consensus
Survey scope	125 respondents from 124 transplant centers in 16 European countries, representing ∼45% of European transplant centers and ∼3,700 living donor transplants annually	​
Kidney function assessment	56% use eGFR (CKD-EPI); 34% use 24-h creatinine clearance; 41% use combined cystatin c and creatinine; 70% use measured GFR (mGFR)	Low consensus/Substantial heterogeneity
GFR thresholds	64% use age-dependent GFR thresholds; 26% of fixed-threshold centers use 80 mL/min/1.73 m^2^	Most centers individualize thresholds according to donor age
Age criteria	63% have no upper age limit; 53% use 18 years as the lower age limit	Age alone is not considered an absolute contraindication
BMI cut-offs	BMI ≥30 kg/m^2^ used by 39%; BMI ≥35 kg/m^2^ by 42%	Obesity management varies considerably across centers
Weight-loss support	74% offer weight-loss interventions, mainly dietary support (67%), endocrinological evaluation/medications 23% and bariatric surgery 11%	Most centers attempt optimization before excluding overweight donors
Diabetes screening	Exclusion thresholds include HbA1c ≥ 53 mmol/mol (46%), fasting glucose ≥7 mmol/L (57%), or OGTT glucose ≥11.1 mmol/L (59%)	Strong consensus on excluding overt diabetes
Hypertension	91% exclude uncontrolled hypertension/end-organ damage; 78% exclude candidates using ≥3 antihypertensives	High consensus regarding hypertension-related exclusions
Albuminuria/ Proteinuria	Only 38% assess both spot and 24-h urine	Marked variability in testing strategies and thresholds
Microscopic hematuria	57% accept donors if both urological evaluation and kidney biopsy are normal	Donation often permitted after negative work-up
ApoL1 testing	27% routinely test donors of African ancestry	ApoL1 testing is not standard practice in most European centers
Nephrolithiasis	Most centers accept low-risk stone history; only 2% reject any history of stones	Stone history is acceptable when recurrence risk is low
Risk calculators	54% do not use online ESKD risk calculators	Clinical judgment remains the primary assessment tool
Informed consent	95% obtain written consent for donation; 65% for data registration	Donation consent is nearly universal
Follow-up duration	83% offer lifelong follow-up, usually annually	Long-term donor surveillance is strongly supported
Follow-up components	Includes blood pressure (98%), eGFR (94%), spot urine (75%), medication review (70%)	Focuses on kidney function and cardiovascular risk

Equally important is protecting potential donors from prolonged evaluation processes and avoiding unnecessary investigations, especially those which are invasive. Unnecessarily prolonged processes can lead to frustration, psychological burden and increase withdrawal rates from donation [9–12]. From the recipient perspective, delays reduce the opportunity to perform transplantation pre-emptively, thereby exposing recipients to dialysis-related morbidity and potentially diminishing the clinical benefits associated with timely LKDT [1, 2]. Incidental findings during the evaluation process, particularly on imaging, are common (reported prevalence range from 6%–75%) [13–15] but most are benign and clinically insignificant, with transmitted donor-derived malignancies being exceedingly rare [16]. Common incidental findings include renal cysts, angiomyolipomas, nephrolithiasis, and microscopic haematuria, for which guideline recommendations are often ungraded due to limited evidence.

Finally, the role of genetic testing in CKD and consequently in living donor evaluation is rapidly evolving. Genetic testing identifies a monogenic cause in ∼10% of adults with CKD and up to 40%–60% in selected groups, often changing diagnosis and management [17, 18]. Targeted genetic testing is most informative in biologically related donors and those with early-onset or unexplained CKD, extrarenal features, or high-risk ancestry (e.g., APOL1). When combined with counseling, it improves risk stratification and donor safety, although challenges remain due to limited guidance, variable expertise, uncertain variant interpretation, access to counseling, and cost.

Over the last decade, living donor selection in several European countries has gradually shifted from fixed exclusion criteria toward more individualised, risk-based assessment. This is now reflected in contemporary guidelines [5] but the impact of these changes is difficult to assess at a European level because donor selection policies, data collection, and follow-up practices remain heterogeneous. Nonetheless, the total number of living donor kidney transplants performed in Europe has increased, exceeding 7,000 procedures across Council of Europe countries in 2024, compared with the mid-2010s when annual numbers were generally reported at around 5000–6000 LDKT [3].

### Donor safety, ethics and outcomes

Ethical dilemmas between potential donors and transplant teams remain common. KDIGO guidelines recommend frameworks to protect donors while recognising that being declined, particularly when labelled “marginal,” may undermine autonomy and cause distress [4, 19–21]. In keeping with contemporary terminology, such candidates are better described as potential donors with expanded criteria. However, respect for donor autonomy does not override the responsibility of transplant teams to prioritise donor safety and clinical judgement.

**FIGURE 2:**
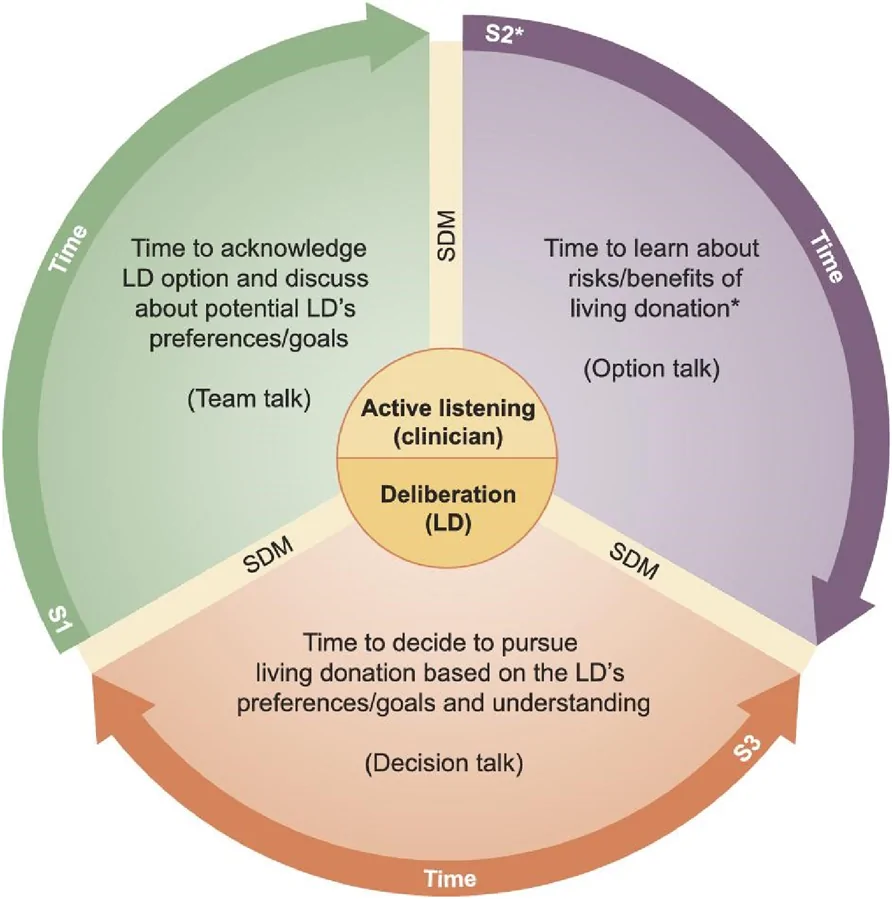
Shared decision-making should start with the primary nephrologist prior to the need for kidney replacement therapy or at the local dialysis unit (S1), and, at the transplant center, with transplant clinicians and surgeons throughout the process of evaluation (S2) and prior to nephrectomy (S3).

Autonomous informed consent requires ensuring donor understanding and freedom from coercion. Transplant programmes should clearly define and communicate how decisions are made and offer second opinions to minimise paternalism [21–25]. A systematic review of informed consent in living kidney donation demonstrated substantial variation across transplant centres and highlighted the need for a standardized yet flexible approach to ensure equitable donor education and decision-making [26]. To address this gap, the DESCaRTES Working Group proposed a 3-Step Model for informed consent in living kidney donation [27], adapted from the Three-Talk Model of shared decision-making [28]. This framework defines consent as a longitudinal process-team, option, and decision talk-standardizing timing and risk communication while allowing individualization based on donor values and understanding (Figure 2).

Living donor evaluation also poses challenges in the interpretation and communication of long-term risk, particularly for the risk of ESKD [29]. Landmark cohort studies by Mjøen et al. and Muzaale et al. demonstrated that, while living kidney donors have a higher relative risk of ESKD compared with healthy controls, the absolute risk remains low (∼0.5% and 0.31% at ∼15 years) [30, 31]. These findings were reinforced by a recent meta-analysis estimating an absolute risk of ESKD of approximately 0.5 cases per 1,000 donor-years. Therefore the attributable risk, which is the excess risk that may be associated with donation itself, remains very small in absolute terms [32]. Interpreting these data requires careful consideration of study design, comparator selection, duration of follow-up, and the extent to which study populations reflect the characteristics of contemporary donor candidates in individual transplant programmes. Healthcare professionals should therefore understand the distinction between relative, absolute, and attributable risk and communicate these concepts clearly to potential donors and recipients, so that risk is interpreted in an accurate and clinically meaningful context.

The high cardiovascular mortality associated with dialysis is well recognised and closely related to the duration of ESKD, with kidney transplantation representing the most effective strategy to reduce this risk [33]. Concerns remain regarding the long-term cardiovascular effects of living kidney donation; however, unlike other unilaterally nephrectomised, often comorbid individuals, living kidney donors generally retain adequate renal functional reserve capacity to support compensatory hyperfiltration [29, 34]. Long-term studies suggest that eGFR trajectories remain stable or improve over time after donation [35], and that eGFR<50 mL/min/1.72m2 is uncommon [36]. Although donors have a higher incidence of hypertension and diabetes, most studies have not demonstrated increased overall mortality or major cardiovascular events [37–42]. A Norwegian cohort reported higher long-term mortality and cardiovascular risk factors, but these findings should be interpreted cautiously given the ethnically homogeneous population, absence of smoking data, and historical donor selection and management practices [30, 43]. Overall, these findings highlight the importance of long-term follow-up, cardiovascular risk reduction, and healthy lifestyle measures after donation.

Women with CKD are at risk of adverse pregnancy outcomes. In living kidney donors, a systematic review found that the risk of preeclampsia increased from 1% to 4%; however, despite the relative increase, the absolute risk remains low and comparable to that of the general obstetric population, with higher BMI and pre-existing hypertension increasing risk [44]. Importantly, there is limited evidence of increased adverse fetal or neonatal outcomes, and post-donation pregnancy has not been associated with accelerated decline in kidney function compared with pre-donation pregnancy. Women who develop gestational hypertension or pre-eclampsia after donation are at increased risk of future hypertension and should receive long-term follow-up [45, 46]. This represents an important opportunity for the transplant community to lead through accurate, evidence-based risk communication. Pregnancy-related risks should be interpreted in the context of study design and individual donor characteristics, with clear distinction between relative and absolute risk. Improved professional education and collaboration with obstetric specialists are essential to ensure balanced counselling and to avoid unnecessarily discouraging suitable women from living kidney donation.

Research on motivations for living kidney donation has identified themes including altruism, family dynamics, and perceived personal benefit [47, 48]. These findings support structured psychosocial screening to identify potential donors who may require additional support, particularly those with limited social support, financial stress, or major caregiving responsibilities. Donors reporting poorer outcomes often described inadequate pre-donation information, highlighting the importance of thorough informed consent, appropriate reassurance and ongoing psychological follow-up [49–52].

### Reducing inequalities

The UK ATTOM study recruited 2055 patients from all 23 transplant centres in the UK and demonstrated that recipients of living donor kidney transplantation were more likely to be young, white, married, and socioeconomically advantaged, with no sex difference in access [53]. Moreover, broader evidence indicates that women are more likely to donate but less likely to receive living donor transplants, particularly in spousal settings independently of HLA factors [54, 55]. Marked ethnic disparities persist, with non-white patients substantially underrepresented among LDKT recipients in both adult and paediatric populations [56–58]. Older patients may be less likely to be referred because of perceived frailty or assumptions regarding limited benefit, while individuals with obesity may face additional barriers related to eligibility criteria and surgical concerns [1, 59–61].

Barriers to LDKT include cultural, geographic, educational, and financial factors, with ethnic minority patients facing additional challenges such as overseas donors, language barriers, and reported discrimination. Improving access requires culturally competent care, including translation services and peer support within transplant programmes [62–64].

Access to LDKT is reduced among less well-educated patients and is closely linked to lower health literacy. Many patient information leaflets and consent documents exceed the average reading age of 11 years [65]. Improving communication, understanding patient perspectives, and using peer educators from diverse communities may help improve engagement and access to living donation. Financial barriers play an important role and may contribute to sex disparities in living donation, as loss of income may disproportionately affect men in some households. Effective reimbursement policies for living donors are therefore essential and economically justified given the long-term cost savings of transplantation over dialysis.

Against this backdrop of inequality, system-level innovations offer opportunities to improve access. Kidney exchange programmes are among the most effective strategies, enabling incompatible donor-recipient pairs to swap donors with other pairs. In Europe, these programmes are typically coordinated by national transplant organisations with multidisciplinary teams responsible for donor-recipient matching, immunological assessment, registry management, logistics, and regulatory oversight. Building on the 2023 Global Convergence in Transplantation recommendation to expand paired donor exchange, the EURO-KEP program was developed, aiming to support national programmes by harmonising eligibility criteria, matching algorithms, and governance frameworks with the goal to establish a pan-European kidney exchange network under common rules [66]. A European exchange programme is considered feasible through stepwise harmonisation of clinical protocols, legal frameworks, interoperable registries, and pilot cross-border collaborations. By enlarging the donor pool, it could improve access for highly sensitised patients and recipients with uncommon blood groups, thereby reducing inequities in LDKT across Europe.

An additional strategy to increase living donation, adopted particularly in the UK and the Netherlands, is unspecified kidney donation to a stranger [67, 68]. Since legalisation in 2006, over 1,000 such transplants have been performed in the UK, with 83 unspecified donors enabling 119 living donor kidney transplants in 2022/23, accounting for 13% of UK living donor transplant activity [69]. Unspecified donation requires careful psychosocial and ethical assessment, robust informed consent, and appropriate management of donor expectations. The BOUnD study showed outcomes comparable to specified donors, without higher rates of withdrawal, regret, or healthcare costs, and estimated that a 10% increase in unspecified donation could save at least £5 million nationally [70]. Long-term Dutch experience has also demonstrated high donor satisfaction [68]. Wider implementation requires experienced multidisciplinary teams, consistent protocols, and greater public and professional awareness [71]. In highly selected cases, unspecified donation has also been performed by individuals with serious life-limiting illnesses or benign renal conditions requiring nephrectomy, illustrating additional opportunities to expand living donation safely and ethically [72, 73].

Highlighting equity in pediatric transplantation is essential. Paediatric access to transplantation in Europe is limited by capacity, variability, and legislation. Addressing these issues requires local initiatives, advocacy, and consideration of broader factors [74]. Care models vary across regions, with differences in demand, surgeon availability, and reliance on mobile or virtual teams. Avoiding ongoing inequity for future generations remains a priority.

### Surgical influences

Minimally invasive surgery can increase donor willingness not only by offering better cosmetic results and quick recovery, but also by increasing safety after donor nephrectomy. Effective surgeon–donor communication and comprehensive informed consent, including clear explanations of risks (e.g., the low mortality rate (<0.01% in the USA, 0.03% in meta-analysis), morbidity 2.3% (intra-op), 7.3% (post-op)) and available surgical options, are essential for improving transplant quality [75, 76]. Most centers still perform hand-assisted laparoscopic donor nephrectomy (57.4%), followed by laparoscopic donor nephrectomy (40.8%) [77]. Retroperitoneal access is underutilized, even though it avoids contact with intra-abdominal organs and colonic mobilization, which can prevent mesenteric or intra-abdominal organ injuries during surgery, as well as adhesions that can cause intestinal obstruction and possible infertility in the long term [78, 79]. Even though several publications show no significant difference between minimally invasive donor nephrectomy surgical techniques, others focus on the benefit of retroperitoneal access in avoiding intra-abdominal complications [80, 81]. Tailored surgical techniques for nephrectomy can reduce barriers and increase safety and satisfaction while expanding donor eligibility for living-donor kidney transplantation. Some examples are: Hand-assisted surgery to increase safety for donors who are obese or have challenging anatomical variations, while donors with low BMI may benefit from a pure laparoscopic or robotic approach. Vaginal extraction of the kidney or single-port techniques can improve cosmesis [82–85]. Retroperitoneal access can be a good alternative to prevent intestinal adhesions for donors with previous abdominal surgery or gastrointestinal motility complaints. Surgeons can be reluctant to perform a right donor nephrectomy because of technical challenges [86]. Hand-assisted retroperitoneal access may also allow more liberal use of the right kidney due to technical advantages, thereby improving donor safety by always preserving the better kidney [78, 87]. Robotic assistance, offering superior precision and ergonomics, may offer benefits over laparoscopy in nephrectomies involving right-sided or complex vasculature donors [88, 89]. The major indication for kidney transplantation with robotic assistance is improved short- and long-term patient and graft survival by decreased wound complications [90]. Avoiding lower abdominal incisions and improved wound outcome is offering a chance for a kidney transplantation without having to first achieve weight loss and therefore, reducing dropout secondary to dialysis-related morbidity and mortality [91, 92]. Robotic approaches are also focused on offering lower analgesic requirements and a shorter learning curve compared to laparoscopic techniques. Enhanced Recovery After Surgery (ERAS) protocols for kidney transplant donors and recipients are also offering important contributions to daily practice. ERAS protocols in kidney transplantation enable optimizing pain control with minimal opioids and promote early mobilization, which have also contributed to faster recovery and increased satisfaction. ERAS offers similar readmission rates, morbidity, and mortality while reducing length of stay, post-operative complications, and postoperative pain [93–95].

### Transplanting incompatible pairs

National opportunities for living donor kidney transplantation for ABO incompatible pairs and highly HLA sensitized patients in Europe differ greatly. Mainly due to a variable access to exchange programs or desensitization strategies.

Although not available everywhere, ABO-incompatible transplantation is widely practiced, with rituximab and antigen-specific immunoadsorption protocols yielding outcomes comparable to ABO-compatible transplants, though infectious complications and higher rejection rates remain concerns [96].

The European Guideline for the Management of sensitized kidney transplant patients proposes a clear approach to a highly sensitized patient [97]. Kidney exchange programs play an important role in this plan.

Successful living donor exchange programs exist on national levels including in the United Kingdom, the Netherlands, South Alliance and Scandiatransplant [98]. In the United Kingdom Living Donor Kidney Sharing Scheme (UKLDKSS) > 70% of the recipients are transplanted making up 23% of the total living donor program. The large number of participants (>250 per run), the inclusion of non-directed altruistic donors (NDAD) and compatible pairs, and allowing ABO incompatible transplants, are, amongst others, contributing to its success. Recent data from the Dutch national kidney exchange programme further support this approach, demonstrating that long-term graft survival after kidney exchange is equivalent to that of direct living donor transplantation, with similar 10-year death-censored graft survival (91.6% vs. 91.9%) [99]. In addition donors participating in KEPs, have similar physical and mental health-related quality of life (HRQoL) outcomes to donors donating directly [100]. These findings support broader adoption of kidney exchange programmes and international collaboration.

Unfortunately, a group of very highly sensitized patients will most probably never receive a graft via these programs and will not be suitable for desensitization strategies. Imlifidase is not available in living donor transplantation. Deceased-donor allocation in acceptable mismatch programs, could offer a solution to these patients yielding long-term graft survival rates comparable to non-sensitized recipients [101], but compromised when compared to LDKT.

Focus on expanding LDKT national exchange programs with more compatible pairs, promoting the participation of NDAD, allowing for ABOi transplantation and by international collaborations (i.e., EURO-KEP), will result in a larger donor pool and a higher chance for the highly sensitized patients to be transplanted in the future [102].

An innovative national strategy in Italy uses deceased-donor initiated chains to overcome immunological incompatibility in living donor kidney transplantation, whereby a deceased-donor kidney enables transplantation for an incompatible pair and triggers a chain of living donor transplants [101]. Early experience demonstrates that, with appropriate ethical oversight and algorithm-based allocation, this approach substantially increases transplant rates among incompatible pairs, reduces waiting times, and improves the efficiency of paired exchange programmes while benefiting waitlisted patients through high-quality chain-ending grafts.

In summary, the integration of risk stratification, advanced antibody testing, individualized allocation strategies and successful and innovative exchange programs are essential to optimize outcomes for HLA and ABO incompatible LDKT pairs across Europe.

### Measuring outcomes in kidney transplantation: New challenges and priorities

The heterogeneous outcomes reported in organ transplant studies prevent comparisons of results across studies and transplant programmes, analysis of international registers and data synthesis in meta-analyses. An increasingly attractive solution that can improve outcome reporting is to use a ‘core outcome set’ (COS), which is an agreed minimum set of outcomes that have to be measured and reported in all studies of a given disease. For example, the Standardized Outcomes in Nephrology-Kidney Transplantation (SONG-Tx) initiative has established a consensus-based COS in kidney transplantation based on the shared priorities of all stakeholders [103, 104].

In the setting of improving short-term transplant outcomes and declining early graft failure, validated surrogate endpoints are increasingly important to evaluate optimization strategies and inform timely clinical and research decisions. The iBox (integrative box) score [105], which combines functional, immunological, and histological parameters to estimate individualized risk of long-term graft loss, has been qualified by the European Medicines Agency as a secondary endpoint for kidney transplant trials and is undergoing further evaluation in a European randomized controlled study [106]. By enabling early assessment of treatment effects and risk stratification, iBox supports personalized post-transplant care and enhances the efficiency of clinical trials [107], with parallel recognition through the FDA biomarker qualification pathway.

A meta-analysis of studies which used machine learning (ML) models in the prediction of kidney graft survival showed that ML-based models had a significantly higher performance than traditional statistical models [107]. ML methods are able to predict delayed graft function using donor maintenance-related variables [108], graft rejection [109] or the development of *de novo* donor-specific antibodies [110]. An unsupervised learning method integrating clinical, immune, and outcome variables revealed five transplant glomerulopathy associated with distinct causes and allograft survival profiles [111]. ML models in transplantation are limited by data quality, representativeness, interpretability, and overfitting, necessitating high-quality datasets, rigorous external validation, fair evaluation across populations, and further consensus to identify the most reliable and generalizable approaches for clinical use.

Europe hosts multiple national and international transplantation registries, and recent initiatives highlight the need for harmonised, high-quality data collection to support robust clinical and epidemiological research. The EDITH project and Council of Europe recommendations advocate for standardized, prospective datasets across all solid organ transplants, leveraging existing electronic systems to improve data completeness, comparability, and analytical value [112, 113]. The European Health Data Space Regulation, effective from March 2025, provides a major opportunity to enable secure, interoperable, and cross-border data sharing while ensuring data protection and patient control. Complementary initiatives, such as the BRAVEST project [114], further underscore the importance of resilient and coordinated data infrastructures. Established systems like Eurotransplant and Scandiatransplant demonstrate how harmonised, interoperable registries can enhance allocation transparency, cross-border collaboration, and ultimately transplant outcomes across Europe.

### Strategies to improve living donation through education and advocacy

From the donor perspective, emotional and psychological barriers often outweigh medical concerns in living donation, including guilt, fear of harm, financial worries, limited knowledge or social networks, and cultural or religious factors. Targeted support and improved education empower patients to engage proactively with potential donors and participate confidently in shared decision-making. Patient organizations help by providing education, support, advocacy, and a sense of community. Established in 2019, the ESOT-ETPO Alliance brings together healthcare professionals and transplant recipients. The ESOT Patient Inclusion Initiative promotes partnership and empowerment to improve knowledge and advocate for patient-centered care [115].

Raising public awareness, building trust among stakeholders, promoting equitable access, removing financial barriers, and encouraging policy changes to improve donation rates, quality, and outcomes for patients and donors drive advocacy initiatives. The European Kidney Health Alliance (EKHA) is a strategic alliance of European non-profit organizations, including patient groups, nephrologists, researchers, and allied health professionals. Its mission is to influence EU health strategies and advocate for harmonized standards of care across Europe [116]. In December 2024, the European Council released conclusions on enhancing organ donation and transplantation, inviting the European Commission to continue work under the proposed action plan to increase donor organ availability and coordinate, promote, and strengthen cooperation between Member States [117]. EKHA and ESOT are working together to achieve this call to action.

Raising awareness about living donor kidney transplantation can be effectively achieved through home outreach programs, like REACH Transplant in the UK [118] and the KRT program in the Netherlands [119]. These initiatives address attendees’ understanding of CKD, facilitate open discussion, support advocacy, and provide signposting to appropriate resources. Data from the Scottish Renal Registry illustrates significant socio-economic disparities in access to pre-emptive transplantation, with the most deprived patients markedly less likely to receive such interventions compared to their more affluent counterparts.

## Conclusion

Living donor kidney transplantation remains the most effective renal replacement therapy for many patients with ESKD, yet access across Europe remains highly inequitable. Key priorities emerging from the symposium include harmonisation of donor assessment and informed consent, stronger donor protection and long-term follow-up, expansion of kidney exchange programmes including cross-border and unspecified donation, and targeted strategies to reduce social, financial, ethnic, and gender-based disparities. Advances in surgical techniques, immunological risk stratification, and digital tools offer important opportunities to improve donor and recipient outcomes but must be implemented within equitable and well-resourced systems of care (Table 2).

**TABLE 2:** Key recommendations, implementation priorities, and future research directions.

Key recommendations	
Domain	Recommendations
A. Harmonise donor evaluation and informed consent	• Develop common minimum standards for donor assessment, risk communication, informed consent, and follow-up • Standardise written information materials and consent processes while allowing individualised donor-recipient decision-making
B. Strengthen donor protection	• Protect donors from unnecessarily prolonged evaluation processes and investigations, particularly invasive, with efficient, timely and well-coordinated assessments • Ensure long-term medical and psychosocial follow-up for all living donors • Prevent financial disadvantage by reimbursing travel, accommodation, childcare, loss of income, and post-donation healthcare costs • Adopt minimally invasive, donor-tailored surgical approaches and enhanced recovery after surgery (ERAS) protocols to improve donor safety, accelerate recovery, and expand access to living donor kidney transplantation
C. Expand kidney exchange programmes	• Support development of national kidney exchange programmes in countries where these are absent or limited • Progressively link national programmes through EURO-KEP or similar frameworks to enable cross-border exchange under shared clinical, legal, ethical, and data-governance standards • Consider developing unspecified kidney donation programmes integrated with kidney exchange schemes and supported by robust psychosocial assessment, informed consent, and multidisciplinary follow-up
D. Reduce inequities in access	• Implement culturally competent education, translation services, peer-support models, and early referral pathways • Prioritise underserved groups, including ethnic minority, socioeconomically disadvantaged, female, paediatric, and geographically remote populations • Promote pre-emptive living donor kidney transplantation through early referral, timely donor evaluation, and targeted interventions to reduce socioeconomic and geographic disparities in access
E. Improve data infrastructure	• Establish harmonised national and European registries for living donor activity, outcomes, and follow-up • Include equity metrics, donor-reported outcomes, and long-term donor safety outcomes in routine data collection
Implementation Priorities	
• Agree on a European core dataset for living donor evaluation and outcomes • Map current reimbursement and donor-protection policies across countries • Expand cross-border kidney exchange between countries with compatible regulatory frameworks • Support countries with low LDKT activity to develop sustainable programmes • Integrate validated risk tools, digital education platforms, and outcome measures into clinical pathways	
Future research directions	
• Define acceptable risk thresholds for expanded donor criteria • Validate donor risk-prediction tools across diverse European populations • Evaluate psychosocial, financial, pregnancy-related, and long-term medical outcomes after donation • Assess the impact of kidney exchange, unspecified donation, genetic testing, digital tools, and minimally invasive surgery on transplant activity, donor safety, and recipient outcomes	

The EURO-KEP programme and development of a pan-European kidney exchange network represent important opportunities to improve equitable access to living donor transplantation. Achieving sustained progress will require collaboration among clinicians, policymakers, patient organisations, and professional societies, supported by legal reform and investment in education and infrastructure. These priorities align with EKITA’s 2026–2027 action plan, which identifies promotion of LDKT as a core European objective.
