# Correction: Landscape of current practices and future perspectives in living donor kidney donation in Europe: proceedings of a pan-European symposium by the European kidney transplant association section of the European Society for Organ Transplantation

**DOI:** 10.3389/ti.2026.17772

**Published:** 2026-09-28

**Authors:** Transplant International Production Office

**Affiliations:** Frontiers Media SA, Lausanne, Switzerland

**Keywords:** cross-border organ exchange, donor evaluation and safety, equity in transplantation, living donor kidney transplantation, transplant registry harmonisation

There was a mistake in the captions of figures 1 and 2 as published. The copyright attributions were incorrect. The corrected captions of figures 1 and 2 appears below.

Figure one: Living donor kidney transplantation rates (per million population) across Europe in 2024. Source: European Directorate for the Quality of Medicines and Healthcare (EDQM). Reproduced from EDQM/Council of Europe and Organización Nacional de Trasplantes (2025), Newsletter Transplant - International figures on donation and transplantation 2024, vol. 30, available at https://go.edqm.eu/nltransplant.

Figure two: FIGURE 2 Shared decision-making should start with the primary nephrologist prior to the need for kidney replacement therapy or at the local dialysis unit (S1), and, at the transplant center, with transplant clinicians and surgeons throughout the process of evaluation (S2) and prior to nephrectomy (S3).

The original version of this article has been updated.

